# Soluble TIM-3 and galectin-9 predict survival in gastric and gastroesophageal junction cancer

**DOI:** 10.1016/j.isci.2025.113871

**Published:** 2025-10-28

**Authors:** David Digomann, Charlotte Reiche, Antonia Stammberger, Tido Willms, Loreen S. Rudek, Anders Grabenkamp, Anna Klimova, Jamie Kölbel, Felix Merboth, Loreen Natusch Bufe, Carolin Beer, Therés Golle, Sarah Cronjaeger, Franziska Hoffmann, Luisa Kranich, Marc Schmitz, Christiane J. Bruns, Hans A. Schlößer, Jürgen Weitz, Lena Seifert, Adrian M. Seifert

**Affiliations:** 1 Department of Visceral, Thoracic and Vascular Surgery, University Hospital Carl Gustav Carus, Technische Universität Dresden, 01307 Dresden, Germany; 2 National Center for Tumor Diseases (NCT), NCT/UCC Dresden, a Partnership Between DKFZ, Faculty of Medicine and University Hospital Carl Gustav Carus, TUD Dresden University of Technology, and Helmholtz-Zentrum Dresden-Rossendorf (HZDR), 01307 Dresden, Germany; 3 German Cancer Consortium (DKTK), Partner Site Dresden, German Cancer Research Center (DKFZ), Heidelberg, Germany; 4 Institute of Immunology, Faculty of Medicine Carl Gustav Carus, Technische Universität Dresden, 01307 Dresden, Germany; 5 Department of General, Visceral, Thorax, and Transplantation Surgery, University of Cologne, Faculty of Medicine and University Hospital Cologne, 50937 Cologne, Germany; 6 Center for Molecular Medicine Cologne, University of Cologne, Faculty of Medicine and University Hospital Cologne, 50937 Cologne, Germany; 7 Center for Integrated Oncology (CIO) Aachen, Cologne and Düsseldorf, Bonn, Cologne 50937, Germany; 8 Institute for Medical Informatics and Biometry, Faculty of Medicine Carl Gustav Carus, Technische Universität Dresden, 01307 Dresden, Germany; 9 Core Unit for Data Management and Analytics (CDMA), National Center for Tumor Diseases (NCT), 01307 Dresden, Germany; 10 Else Kröner Clinician Scientist Professor for Translational Tumor Immunological Research, 01307 Dresden, Germany

**Keywords:** Immunology, Cancer, Transcriptomics

## Abstract

Current biomarkers for diagnosis and prognosis prediction in gastric cancer (GC) and gastroesophageal junction (GEJ) cancer have limited accuracy, and the role of soluble immune checkpoints is unknown. In this study, T cell immunoglobulin and mucin domain-containing-3 (TIM-3) and galectin-9 expression was investigated in single-cell RNA (scRNA) sequencing data and with multiplex immunohistochemistry. Further, serum samples from 310 patients with GC/GEJ and 82 healthy donors were analyzed for soluble TIM-3 (sTIM-3) and galectin-9 (sGal-9). sGal-9 levels were significantly increased in patients with cancer compared to healthy donors (*p* < 0.001). Notably, the combination of sTIM-3 and sGal-9 had a higher diagnostic accuracy for GC/GEJ than the established tumor markers CEA, CA19-9, and CA72-4. Patients with GC/GEJ with increased sTIM-3 and sGal-9 levels had a significantly reduced overall survival with a hazard ratio of 1.77 (sTIM-3 high), 1.82 (sGal-9 high), and 2.27 (sTIM-3 high+sGal-9 high), respectively*.* Collectively, sTIM-3 and sGal-9 may serve as novel non-invasive biomarkers for diagnosis and outcome prediction in patients with GC/GEJ.

## Highlights

• TIM-3 and galectin-9 are expressed in gastric and gastroesophageal junction cancer
• Patients with GC/GEJ have increased sGal-9 serum levels compared to healthy individuals
• High serum levels of sTIM-3 and sGal-9 are associated with reduced overall survival
• sTIM-3 and sGal-9 are novel diagnostic and prognostic biomarkers

## Graphical abstract

**Figure:**
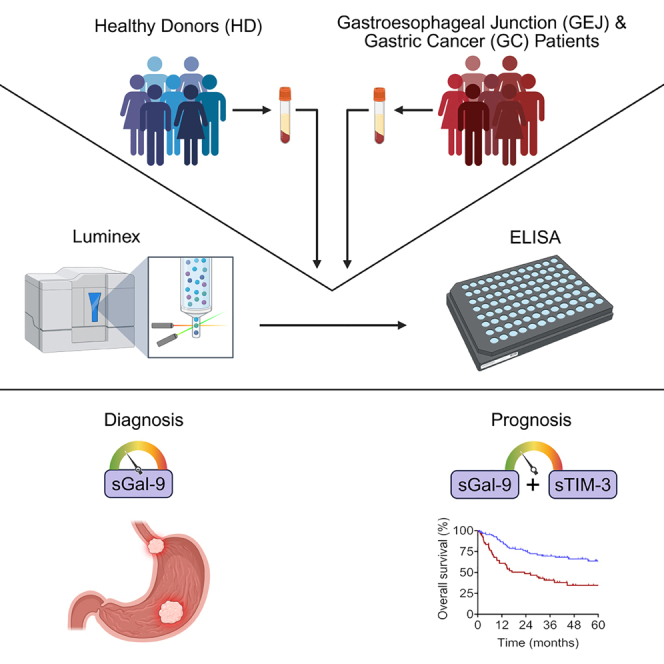

## Introduction

Immunotherapy has been established as another pillar of cancer treatment. Results from the multinational, double-blind, randomized phase 3 trial MATTERHORN, confirmed the benefits of PD-L1 blockade via durvalumab in addition to perioperative chemotherapy in gastric cancer (GC) and cancer of the gastroesophageal junction (GEJ).^1^ However, upregulation of alternative immune checkpoints can cause resistance to PD-L1 blockade. One important pathway is mediated via T cell immunoglobulin and mucin domain-containing-3 (TIM-3).^2^ TIM-3 is an immune checkpoint receptor that has emerged as an important player in the modulation of immune responses.^3^ TIM-3 was first described in 2002 as a marker of interferon-γ-producing CD4^+^ and CD8^+^ T cells.^4^ TIM-3 is expressed on a variety of immune cell populations, like regulatory T cells (Tregs),^5^ natural killer (NK),^6^ B,^7^ as well as myeloid^8^ and mast cells.^9^ It is commonly described as a co-inhibitory receptor. However, as its cytoplasmic tail lacks known inhibitory signaling motifs, the exact mechanism is still under investigation. A soluble isoform of TIM-3 (sTIM-3) can be generated either through alternative splicing at the mRNA level, as reported in murine models,^10^ or via proteolytic cleavage of the membrane-bound protein by the matrix metalloproteinases ADAM10 and ADAM17.^11^ If not bound to a ligand, TIM-3 interacts with human leukocyte antigen-B-associated transcript 3 and maintains T cell activation.^12^ Under conditions of short-term antigen stimulation, TIM-3 was described to augment T cell activation although it is also associated with T cell exhaustion.^13^ The diverse expression pattern of TIM-3 and its ligand-depending effector potential highlights the crucial impact of its molecular interactions. The first described and most studied ligand is galectin-9 (Gal-9).^14^ As a member of the galectin family of β-galactoside-binding proteins, Gal-9 is mainly found in the cytoplasm of cells in various tissues. Its secretion in its soluble form is described as a non-classical pathway.^15^^,^^16^^,^^17^ Initially characterized by its ability to promote cell aggregation and adhesion through crosslinking glycoproteins forming gal-glycoprotein grids, Gal-9 has been implicated in a broad variety of biological processes, for example, modulating cell signaling, apoptosis, immune cell migration, and cytokine secretion.^18^ In GC, Gal-9 has both pro- and anti-tumor effects that reflect the complexity of its functions.^19^^,^^20^ Gal-9 can promote immune evasion by inducing T cell death of terminally exhausted TIM-3-positive T cells impairing anti-tumor immunity.^21^^,^^22^ However, it can also act as a tumor suppressor by modulating tumor cell adhesion, inhibiting metastasis, and inducing apoptosis in certain cancer cells.^23^^,^^24^ Decreased Gal-9 and increased TIM-3 protein expression in the tumor microenvironment were associated with poor prognosis in GC.^24^ The clinical relevance of their soluble forms is unknown in GC and GEJ. In our discovery cohort, we found sTIM-3 as a putative prognostic marker in GC and GEJ (Figures S3A and S3B). Our study evaluated and validated the diagnostic and prognostic value of sTIM-3 and soluble Gal-9 (sGal-9).

## Results

### TIM-3 and Gal-9 are expressed in human GC

**Figure 1:**
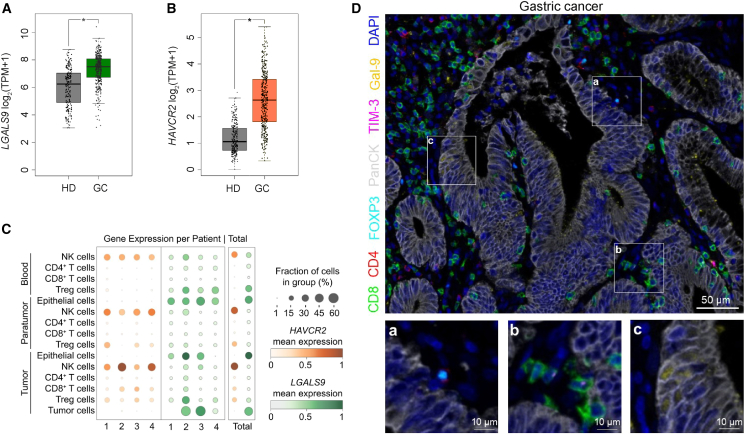
TIM-3 and Gal-9 are expressed in human GC Gene expression of (A) *LGALS9* and (B) *HAVCR2* in gastric cancer (GC) compared to expression in healthy controls (HD). Results are displayed in log2 of transcripts per million (TPM+1) using boxplots with jitter. Data was obtained from TCGA and GTEx projects. Results were processed with the GEPIA web service. GC, *n* = 408; HDs *n* = 211. ∗, *p* < 0.05. (C) Mean mRNA expression and the fraction of *HAVCR2* or *LGALS9*-positive cells in different cell types of three compartments (blood, paratumor, and tumor). Four patients with GC and their averages were analyzed. Minimal and maximal expression was normalized for data of each rectangle separately. (D) Paraffin-embedded human GC specimens were stained for PanCK (gray), CD4 (red), CD8 (green), FOXP3 (cyan), TIM-3 (purple), and Gal-9 (yellow). A representative multiplex immunofluorescence image is shown (scale bars as indicated).

First, mRNA levels of *HAVCR2* (TIM-3) and *LGALS9* (Gal-9) were investigated in GC (*n* = 408) and healthy stomach tissue samples (*n* = 211) from The Cancer Genome Atlas (TCGA) database. Both *HAVCR2* and *LGALS9* expression levels were significantly higher in tumor compared to normal tissue (Figures 1A and 1B). Single-cell sequencing data were obtained, and four patients with GC were analyzed. After different levels of clustering, cell types of interest were identified, and the expression of *LGALS9* and *HAVCR2* was retrieved for each cell type and different compartments (blood, paratumor, and tumor tissue). The most prominent expression of *HAVCR2* was observed in NK cells in all compartments followed by tumor-infiltrating lymphocytes, mainly CD8^+^ T cells and Tregs. *LGALS9* was expressed in Tregs, CD4^+^ T cells, and NK cells in the blood compartment. Notably, *LGALS9* expression in para- and tumor tissue was predominantly detected in epithelial cells and in tumor cells in particular (Figures 1C, S1A, S1D, S2A, and S2F). Multiplex immunohistochemistry staining was performed to further analyze the protein expression and cell-specific distribution of TIM-3 and Gal-9 in GC. TIM-3 was expressed by CD8^+^ T cells, while Gal-9 was mostly expressed by panCK-positive tumor cells (Figure 1D).

### sTIM-3 and sGal-9 serum levels detect GC/GEJ

**Figure 2:**
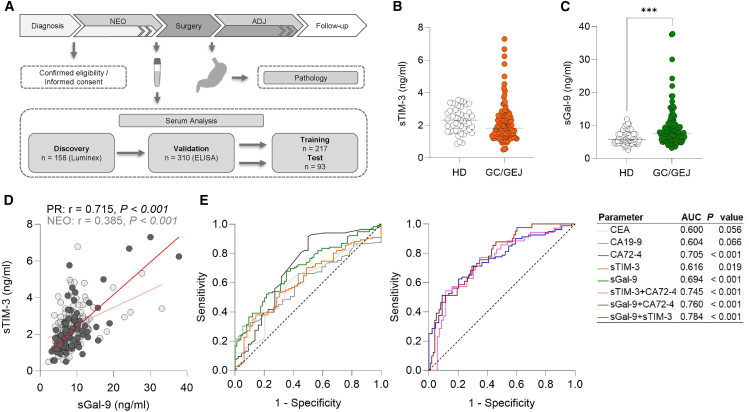
sTIM-3 and sGal-9 serum levels detect GC/GEJ (A) Schematic overview of the study workflow. (B) Serum levels of sTIM-3 from patients with GC/GEJ and HD with median (unpaired *t* test with Welch’s correction, HD vs. GC/GEJ *p* = 0.32). (C) Serum levels of sGal-9 from patients with GC/GEJ and HD with median (unpaired *t* test with Welch’s correction, HD vs. GC ∗∗∗, *p* < 0.001). (D) Scatterplot with Pearson correlation of sTIM-3 and sGal-9 serum levels from patients with primarily resected GC/GEJ (*n* = 98). (E) ROC of sTIM-3, sGal-9, CEA, CA19-9, and CA72-4 with an AUC significantly different from chance of CA72-4, sTIM-3, and sGal-9 (CEA: *n* = 44/101 [HD/GC/GEJ]; confidence interval [CI]: 0.501–0.699; CA19-9: *n* = 37/89 [HD/GC/GEJ]; CI: 0.505–0.703; CA72-4: *n* = 44/84 [HD/GC/GEJ]; CI: 0.603–0.806; sGal-9: *n* = 74/115 [HD/GC/GEJ]; CI: 0.620–0.769). ROC of sTIM-3 and sGal-9 as combined markers based on logistic regression analysis with an AUC significantly different from chance for all combinations (sTIM-3+CA72-4: *n* = 35/69 [HD/GC/GEJ]; CI: 0.645–0.845; CI: 0.639–0.820; sGal-9+CA72-4: *n* = 44/80 [HD/GC/GEJ]; CI: 0.676–0.844; sGal-9+sTIM-3: *n* = 41/98 [HD/GC/GEJ]; CI: 0.705–0.863).

sTIM-3 serum level was determined in a discovery cohort of 158 patients with GC/GEJ using Luminex technology (Figures S3A and S3B). Samples were further validated using enzyme-linked immunosorbent assay. Additional samples were obtained, and sGal-9 serum levels were determined concordantly to sTIM-3 in this cohort of 310 patients with GC/GEJ. For cutoff investigations, patients were randomly subdivided into a training (*n* = 217) and test (*n* = 93) cohort (Figure 2A). The serum levels were compared to healthy donors (HDs; *n* = 82). While sTIM-3 was not increased (Figure 2B), the level of sGal-9 was significantly higher in the serum of patients with GC/GEJ compared to HDs (Figure 2C). A potential correlation of sTIM-3 and sGal-9 was investigated, providing a Pearson correlation coefficient (r) of 0.715 (Figure 2D). Further, the serum levels of both proteins were compared to the tumor markers carcinoembryonic antigen (CEA), cancer antigen 72-4 (CA72-4), and carbohydrate antigen 19-9 (CA19-9) as a binary classifier and plotted as receiver operating characteristic (ROC) curve. sGal-9 levels were able to discriminate between HDs and patients with GC/GEJ (area under the curve [AUC] 0.694), whereas the other markers showed the same or inferior capabilities of discrimination (AUC for CEA: 0.6; CA72-4: 0.705; and CA19-9: 0.604; Figure 2E). An optimized cutoff value determined by Youden’s J statistic was detected at a serum concentration of 6.5 ng/mL for sGal-9 in the training cohort. Application toward the test cohort demonstrated a sensitivity of 72% and a specificity of 65% (Table S1). Consequently, the combinational potential of both proteins as diagnostic markers was investigated through logistic regression with combined classifiers. Strikingly, sTIM-3 and sGal-9 serum levels together outperformed any of the tested single marker to discriminate HDs from patients with GC/GEJ (AUC: 0.784, 91.8% sensitivity, and 41.5% specificity), while the combination of sGal-9 or sTIM-3 with another tumor marker was inferior (Figures 2E and S4A; Table S2).

### sTIM-3 and sGal-9 levels are independent of tumor stage in GC/GEJ

**Figure 3:**
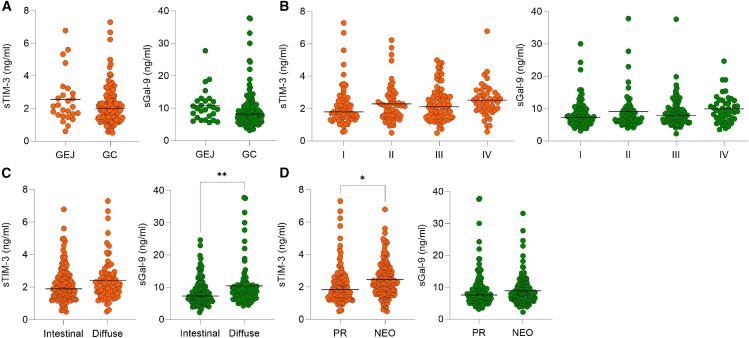
sTIM-3 and sGal-9 levels are independent of tumor stage in GC/GEJ (A) Serum levels of sTIM-3 and sGal-9 in GC/GEJ with median (sTIM-3: *p =* 0.33, sGal-9: *p =* 0.26). Two-tailed unpaired *t* test was applied. (B) Serum levels of sTIM-3 and sGal-9 for different UICC stages presented with median. sTIM-3: UICC I vs. UICC II, *p =* 0.941; UICC I vs. UICC III, *p =* 0.887; UICC I vs. UICC IV, *p =* 0.395; UICC II vs. UICC III, *p =* 0.999; UICC II vs. UICC IV, *p =* 0.727; UICC III vs. UICC IV, *p =* 0.762. sGal-9: UICC I vs. UICC II, *p* = 0.902; UICC I vs. UICC III, *p* = 0.989; UICC I vs. UICC IV, *p* = 0.41; UICC II vs. UICC III, *p* = 0.976; UICC II vs. UICC IV, *p* = 0.813; UICC III vs. UICC IV, *p* = 0.544. One-way ANOVA using Tukey’s multiple comparisons tests. (C) Serum levels of sTIM-3 and sGal-9 in GC/GEJ patients with different histologic types according to Lauren classification, with median. sTIM-3: intestinal vs. diffuse, *p =* 0.14; sGal-9: intestinal vs. diffuse ∗∗, *p =* 0.005. Two-tailed unpaired *t* test. (D) Serum levels of sTIM-3 and sGal-9 compared between GC/GEJ patients treated with primary resection (PR) or neoadjuvant chemotherapy (NEO) with median. sTIM-3: PR vs. NEO ∗, *p =* 0.03; sGal-9: PR vs. NEO, *p =* 0.706. Two-tailed unpaired *t* test.

We investigated the association of sTIM-3 and sGal-9 serum levels with tumor location, tumor stage, Lauren histologic type, and treatment status. Patients with GEJ and GC had similar serum levels of both soluble immune checkpoints (Figure 3A; Tables S5 and S6). sTIM-3 and sGal-9 were increased with tumor stage but without statistical significance (Figure 3B). Tumors with an intestinal or diffuse histological type according to the Lauren classification showed significant differences in sGal-9 levels (Figure 3C). Notably, patients who underwent neoadjuvant chemotherapy prior to surgery showed increased serum levels for sGal-9 and sTIM-3 compared to patients who underwent primary resection (PR) with a statistically significant increase in sTIM-3 levels (Figure 3D).

### High levels of sTIM-3 and sGal-9 predict reduced overall survival in patients with GC/GEJ

**Figure 4:**
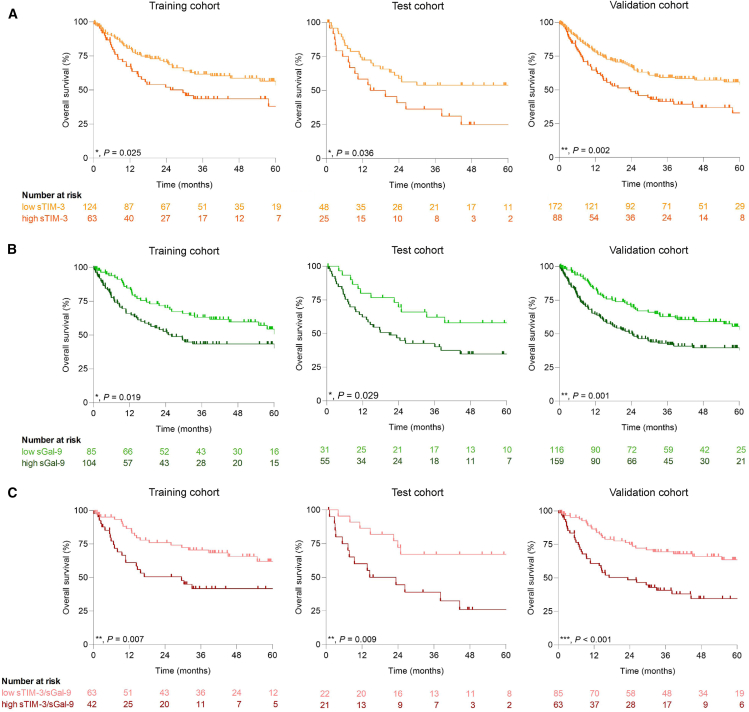
High levels of sTIM-3 and sGal-9 are associated with reduced overall survival in patients with GC/GEJ (A) Kaplan-Meier curves of GC/GEJ patients with high or low sTIM-3 levels in the training, test, and validation cohorts. A threshold of 2.5 ng/mL for sTIM-3 was applied. Median survival of sTIM3 low vs. high in training: undefined∗ vs. 29.52 months, log rank *p =* 0.025; test: undefined∗ vs. 17.02 months, log rank *p =* 0.036; validation: undefined∗ vs. 23.8 months, log rank *p =* 0.002; *∗* probability of survival exceeds 50% at the longest time point. (B) Kaplan-Meier curves of GC/GEJ patients with high or low sGal-9 levels in the training, test, and validation cohort. A threshold of 7.2 ng/mL for sGal-9 was applied. Median survival of sGal-9 low vs. high in training: undefined∗ vs. 25.95 months, log rank *p =* 0.019; test: undefined∗ vs. 20.88 months, log rank *p =* 0.029; validation: undefined∗ vs. 24.41 months, log rank *p =* 0.001; *∗* probability of survival exceeds 50% at the longest time point. (C) Kaplan-Meier curves of GC/GEJ patients with high or low sTIM-3 and sGal-9 levels in the training, test, and validation cohort. The aforementioned thresholds (2.5 and 7.2 ng/mL) were applied. Median survival of sTIM-3/sGal-9 low vs. high in training: undefined∗ vs. 29.52 months, log rank *p =* 0.007; test: undefined∗ vs. 18.92 months, log rank *p =* 0.009; validation: undefined∗ vs. 23.31 months, log rank *p <* 0.001; *∗* probability of survival exceeds 50% at the longest time point.

To further determine the prognostic value of sTIM-3 and sGal-9, an empirical approach with Cox regression was used to determine thresholds in the training cohort, which were then applied to the test, and the validation cohort (Table 1). This resulted in the most distinct hazard ratio (HR) for patients with sTIM-3 levels of >2.5 ng/mL and sGal-9 >7.2 ng/mL, respectively. Kaplan-Meier survival curves showed a significantly reduced overall survival for patients with high sTIM-3 and high sGal-9 levels (HR for sTIM-3 1.746 in training, 2.115 in test, and 1.770 in validation cohort; and for sGal-9 1.676, 1.938, and 1.824, respectively; Table 2; Figures 4A and 4B). To rule out clinicopathological factors as confounders and to put sTIM-3 and sGal-9 into relation to other tumor markers, a multivariable Cox regression analysis was performed, confirming a significantly higher HR for patients with high sGal-9 levels (Table 3). Survival analysis for NEO and PR GC/GEJ with both markers revealed an increased prognostic relevance for the PR subpopulation (Figures S5A and S5B). Based on the possibilities of combining both markers for diagnosis, we investigated their combined relevance for prognosis. Patients with GC/GEJ with levels of sTIM-3 >2.5 ng/mL and sGal-9 >7.2 ng/mL compared to patients below these thresholds had HRs of 2.441 in the training, 3.225 in the test, and 2.268 in the validation cohort, respectively (Table 2; Figure 4C), underlining their role as prognostic biomarkers.

**Table 1:** Clinicopathologic characteristics of the study cohorts

Cohort	Validation, *n* (%)		Training, *n* (%)		Test, *n* (%)		*p* value^a^
Total	310		217		93		–
Mean age	63.49 (years)		61.35 (years)		63.67 (years)		0.874^a^

**Sex**							

Female	125	(40.32)	91	(41.94)	34	(36.56)	0.449^b^
Male	185	(59.68)	126	(58.06)	59	(63.44)	–

**Tumor localization**							

GEJ	33	(10.65)	18	(8.29)	15	(16.13)	**∗0.046**^b^
Stomach	277	(89.35)	199	(91.71)	78	(83.87)	–

**T stage**							

0	17	(5.48)	15	(6.91)	2	(2.15)	0.52^c^
1	62	(20)	42	(19.35)	20	(21.51)	–
2	31	(10)	23	(10.6)	8	(8.6)	–
3	111	(35.81)	73	(33.64)	38	(40.86)	–
4	75	(24.19)	54	(24.89)	21	(22.58)	–
Unknown	14	(4.52)	10	(4.61)	4	(4.3)	–

**N stage**							

0	125	(40.32)	84	(38.71)	41	(44.09)	0.356^c^
1	47	(15.16)	36	(16.59)	11	(11.83)	–
2	46	(14.84)	28	(12.9)	18	(19.35)	–
3	77	(24.84)	58	(26.73)	19	(20.43)	–
Unknown	15	(4.84)	11	(5.07)	4	(4.3)	–

**M stage**							

0	257	(82.9)	180	(82.95)	77	(82.8)	>0.999^b^
1	53	(17.1)	37	(17.05)	16	(17.2)	–

**UICC stage**							

CR	1	(3.03)	15	(6.91)	3	(3.22)	0.792^c^
I	7	(21.21)	53	(24.43)	24	(25.81)	–
II	7	(21.21)	47	(21.66)	22	(23.66)	–
III	6	(18.18)	65	(29.95)	28	(30.11)	–
IV	12	(36.37)	37	(17.05)	16	(17.2)	–

**Neoadjuvant treatment**							

Yes	178	(57.42)	123	(56.68)	55	(59.14)	0.91^b^
No	128	(41.29)	91	(41.94)	37	(39.78)	–
Unknown	4	(1.29)	3	(1.38)	1	(1.08)	–

∗Training vs. test

a *t* test

b Fisher’s exact test

c Chi-squared test

**Table 2:** Univariate Cox regression analysis

	HR (95% CI)	*p* value	*n*
**sTIM-3**			

Low vs. high	1.77 (1.22–2.567)	**∗∗0.003**	260

**sGal-9**			

Low vs. high	1.824 (1.268–2.625)	**∗∗0.001**	276

**sTIM-3+sGal-9**			

Low vs. high	2.268 (1.447–3.556)	**∗∗∗<0.001**	184

**Table 3:** Multivariate Cox regression analysis (*n* = 215)

	HR (95% CI)	*p* value
**Age**		

Years	1.007 (0.988–1.025)	0.494

**Sex**		

Female vs. male	0.861 (0.553–1.341)	0.508

**T**		

1 vs. 2	0.677 (0.231–1.981)	0.476
1 vs. 3	1.181 (0.545–2.564)	0.673
1 vs. 4	1.5 (0.644–3.497)	0.347

**N**		

0 vs. 1	0.932 (0.437–1.988)	0.856
0 vs. 2	1.55 (0.727–3.306)	0.257
0 vs. 3	3.384 (1.711–6.693)	**∗∗∗<0.001**

**M**		

0 vs. 1	2.386 (1.427–3.99)	**∗∗∗<0.001**

**Serum marker**		

CEA	0.979 (0.935–1.025)	0.363
CA72-4	1.004 (0.998–1.009)	0.166
CA19-9	1.001 (1–1.001)	0.073
sTIM-3 low vs. high	1.262 (0.804–1.981)	0.312
sGal-9 low vs. high	1.841 (1.137–2.983)	**∗0.013**

## Discussion

GC and GEJ are among the ten cancers with the highest incidence and mortality and show an increasing worldwide public health concern.^25^ GC is estimated to increase to 1.8 million new cases and to be responsible for 1.3 million deaths per year by 2040, which corresponds to an increase of 66% and 71%, respectively, compared to 2020.^26^ Worldwide, the average 5-year survival rate is approximately 23%.^27^ Late diagnosis and consecutive advanced-stage tumors at presentation are considered the leading cause of this unfavorable prognosis. Endoscopic examination with biopsies is current practice for diagnosing GC.^28^ In East Asian countries with a high incidence of GC, screening programs based on endoscopy have been established. These initiatives have led to increased detection of early-stage GC and significantly reduced mortality rates.^29^ Conversely, in countries with a low GC incidence, routine endoscopic screening for asymptomatic individuals is not recommended, taking the patient risk and cost associated with this examination into account. However, due to the expected increase in patients with GC, experts recommended population-based prevention programs also in low-risk countries.^30^^,^^31^ Accordingly, a less invasive and cheaper preselection tool with high sensitivity should be considered. Tumor markers like CEA, CA19-9, or CA72-4 are frequently measured in patients with GC, but their application is controversial.^28^^,^^32^ In this study, the combination of sTIM-3 and sGal-9 had a sensitivity of 92% with a specificity of 42% to discriminate patients with GC and GEJ from healthy individuals in a logistic regression model applied to our cohort. However, our data revealed no difference in sTIM-3 levels between GC/GEJ patients and HDs, different from other tumor entities.^33^ Here, we show that TIM-3 is mainly expressed on immune cells, while Gal-9 is expressed by tumor cells. Thus, sGal-9 may be associated with the presence of cancer cells, while sTIM-3 is associated with cancer-related immune exhaustion. GC has a less exhausted immune tumor microenvironment compared to other tumors,^34^ which may reflect the similar level of sTIM-3 in HDs and GC/GEJ and the good discrimination of both cohorts by sGal-9. Overall, while sTIM-3 alone has no diagnostic relevance, both markers combined may serve as tumor markers and as a preselection tool for further diagnostics.^29^

In addition to the need for improved early diagnosis, current methods for patient stratification—including prognosis prediction, surgical assessment, postoperative treatment decisions, and follow-up planning based on clinicopathological staging—are increasingly variable and lack the precision required for modern therapies. Almost all approved therapies are based on marker stratification. For example, trastuzumab, an antibody targeting HER2, is used in combination with chemotherapy for patients with HER2-positive advanced GC or GEJ.^35^ Nivolumab, an anti-PD-1 antibody, was recently proven to be beneficial in combination with chemotherapy as first-line therapy in advanced or metastatic GC, GEJ, and esophageal adenocarcinoma.^36^^,^^37^ Various studies have highlighted the crucial role of soluble immune checkpoint modulators in immunotherapy, either through direct interaction with their corresponding receptors or ligands, by indirectly altering the targeted effector cells, or by neutralizing the immunomodulatory drugs through binding.^38^^,^^39^^,^^40^

Consistent with our findings, a previous study identified a correlation between advanced tumor stage, poor prognosis, and a high percentage of TIM-3-positive CD4^+^ and CD8^+^ T cells indicating immune cells as a potential cellular source for its soluble form. In contrast, Gal-9 expression on tumor cells was inversely associated with tumor stage and prognosis, while Gal-9 tumor expression was high compared to normal tissue. The high expression within the tumor and by tumor cells is consistent with our data and points toward tumor cells as source of its soluble form.^24^^,^^41^ In hepatocellular carcinoma, high sGal-9 levels were associated with improved survival. However, tissue expression did not correlate with serum levels, supporting the missing association between tumor stage and serum concentrations in our study.^42^ Overall, further investigations are needed to verify the association between tissue and serum levels of these markers.

Interestingly, a recent study demonstrated that patients with cholangiocarcinoma and non-small cell lung cancer unresponsive to anti-PD-1 therapy had significantly higher sTIM-3 levels. Metalloprotease ADAM10/17 is described to cleave TIM-3 and generate sTIM-3, which binds to Caecam1, pushing effector T cells into an exhausted phenotype resistant to anti-PD-1 treatment. Inhibiting antibodies of ADAM10/17, CAECAM, or TIM-3, neutralizing sTIM-3, may reinvigorate the T cell response, particularly in anti-PD-1-resistant tumor patients.^40^ Although these results need further validation, this underscores the previously mentioned need for more data of soluble immune checkpoints in various cancers, especially since several anti-PD-1 and other immunotherapy trials are ongoing in GC/GEJ, and multiple clinical trials targeting TIM-3 or Gal-9 in various cancer types are underway.^1^^,^^43^^,^^44^ In this context, the Gal-9-inhibiting antibody LYT-200 was recently granted fast-track development by the Food and Drug Administration in patients with recurrent or metastatic head and neck squamous cell carcinoma. In our study, patients with high sTIM-3 and or high sGal-9 serum levels showed a significantly reduced overall survival. Combining both markers could further increase their prognostic potential and underlines their putative role in stratification, especially in the context of immunotherapies. Since sTIM-3 and sGal-9 levels showed a strong correlation, but the range of sGal-9 was much higher than the range of sTIM-3, the multivariate analysis determined only sGal-9 as an independent survival predictor. Notably, age was not an independent factor, despite the sGal-9- and sTIM-3-high cohorts being significantly older than the protein-low cohorts. To further rule out a confounding role of age, cutoff optimization and validation were repeated using a multivariate Cox regression model including age as a further factor. The HR for age was between 0.984 and 1.013 with only significant independence in one of the three cohorts. To rule out age as a confounder of the diagnostic capabilities, a propensity score matching for HDs and GC was performed, and discrimination analyses were repeated in cohorts without differences in age (Figures S4B–S4D). Overall, correlation analyses showed a weak correlation between age and serum levels for both markers, without clinical relevance (Figures S4E and S4F).

Besides age, the sGal-9 level was higher in diffuse than intestinal GC, while sTIM-3 levels were higher in patients after neoadjuvant chemotherapy compared to patients who were resected without prior treatment. Collectively, sGal-9 may serve as a new diagnostic biomarker, and the combination of sTIM-3 and sGal-9 has high prognostic relevance, which may enable treatment stratification for patients with GC/GEJ in the future.

### Limitation of this study

Most patients in our study underwent surgical resection with a curative intent (87%), not representing the general GC/GEJ population. In survival analyses, both markers showed a slightly weaker prediction capability in neoadjuvant-treated patients. To translate these findings for neoadjuvant treatment approaches, further analyses of sGal-9 and sTIM-3 levels over the course of treatment are needed. Additionally, as sTIM-3 and sGal-9 are increased in the serum of patients with other tumor types, their specificity for GC and GEJ is limited.

## Resource availability

### Lead contact

Further information and requests for resources should be directed to and will be fulfilled by the lead contact, Adrian M. Seifert, MD (adrian.seifert@ukdd.de).

### Materials availability

This study did not generate new unique reagents. Serum and tissue samples are not publicly available due to their usage agreement.

### Data and code availability

• Raw data are available from the corresponding author upon reasonable request. Single-cell sequencing data (.fastq files) were accessed through the Genome Sequencing Archive from the China National Center for Bioinformation under the accession number HRA000704. The sequencing data were made public through the publication by Sun et al.^45^ Information for sample collection, processing, and library preparation can be accessed through the linked paper in the STAR Methods section.
• The codes used for the single-cell sequencing data analyses can be accessed here: https://github.com/TidoWillms94/Paper-with-Code-scRNAseq-GC.
• Any additional information required to reanalyze the data reported in this paper is available from the lead contact upon request.

## Acknowledgments

This work was supported by the 10.13039/100014435Federal Ministry of Education and Research and co-funded by the 10.13039/501100000780European Commission (01KT2304B; M.S.), the 10.13039/100014435Federal Ministry of Education and Research (03ZU1111LB; M.S.), the 10.13039/501100007466Ernst-Jung Stiftung (L.S.), the 10.13039/100007651German Research Foundation (DFG; SE2980/5-1; L.S.), the 10.13039/501100003042Else Kröner-Fresenius-Stiftung (Else Kröner Clinician Scientist Professorship; L.S.), the 10.13039/501100007422Monika Kutzner Stiftung (A.M.S.), the German Cancer Consortium (10.13039/501100012353DKTK; A.M.S.), and the Federal Ministry of Education and Research (10.13039/501100002347BMBF; Advanced Clinician Scientist Program CAMINO Dresden; A.M.S.). We are grateful to Heike Polster for the assistance with the acquisition of patient serum and excellent technical support, as well as to the German Red Cross Blood Donation, especially to Corinna Opitz for her help and technical support in operating the Luminex machine at the German Red Cross Blood Donation Service North-East. The flow chart and the graphical abstract were created with BioRender.org.

## Author contributions

D.D., A.M.S., and L.S. did the conceptualization. D.D. and C.R. performed the clinical and experimental data collection. A.S. and M.S. performed the staining experiments, and T.W. conducted the single-cell sequencing analyses. L.S.R., A.G., C.J.B., and H.A.S. provided resources and supported the investigation. Statistical advice was provided by A.K. Figure design was supported by J.K. D.D. wrote the manuscript with input from A.M.S. and L.S. Funding and project administration were provided by L.S. and A.M.S. J.W. provided the research facilities. All authors reviewed the manuscript.

## Declaration of interests

H.S. received funding for research from AstraZeneca and Tabby Therapeutics.

## STAR★Methods

### Key resources table

**Table:** 

REAGENT or RESOURCE	SOURCE	IDENTIFIER
**Antibodies**		

Anti-CD4	Abcam plc.	EPR6855; RRID:AB_2750883
Anti-CD8	Ventana Medical Systems, Inc.	SP57; RRID:AB_2335985
Anti-FoxP3	Abcam plc.	A236A/E7; RRID:AB_445284
Anti-Gal-9	Cell Signaling Technology, Inc.	D9R4A; RRID:AB_2799456
Anti-PanCK	Ventana Medical Systems, Inc.	AE1/AE3/PCK26; RRID:AB_2810237
Anti-TIM-3	Cell Signaling Technology, Inc.	D5D5R; RRID:AB_2716862

**Biological samples**		

Serum sample *n* = 175	Biobank, University hospital Dresden	NA
Serum sample *n* = 135	Biobank, University hospital Cologne	NA

**Critical commercial assays**		

MILLIPLEX Human Immuno-Oncology Checkpoint Protein Panel	Merck Millipore	HCKP1-11K
Human TIM-3 ELISA Kit – Quantikine	R&D Systems	DTIM30
Human Gal-9 ELISA Kit – Quantikine	R&D Systems	DGAL90

**Deposited data**		

GEPIA	Tang et al.^25^	RRID:SCR_026154
Single-cell sequencing data	Genome Sequencing Archive from the China National Center for Bioinformation (CNCB); K. Sun et al.^30^	Accession number: HRA000704

**Software and algorithms**		

R statistical software (Version 4.2.0)	R Foundation for Statistical Computing	RRID:SCR_001905
GraphPad Prism 9.0	GraphPad Software	RRID:SCR_002798
Cell Ranger 9.0.0	10x Genomics	RRID:SCR_017344
Scanpy 1.10.4	Scanpy development team	RRID:SCR_018139
scDblFinder 1.16.0	Bioconductor	RRID:SCR_022700
Phenochart™ software	Akoya Biosciences	RRID:SCR_019156
inForm software	Akoya Biosciences	RRID:SCR_019155
ImageJ software	Wayne Rasband	RRID:SCR_003070

### Experimental model and study participant details

#### Patient samples

Serum samples from 310 patients diagnosed with GC or GEJ (GC *n* = 277; GEJ Siewert type I/II, *n* = 3; GEJ Siewert type III, *n* = 30), who underwent surgical treatment at the Department of General, Visceral, Thorax, and Transplantation Surgery at the University Hospital Cologne (Cologne, Germany) or the Department of Visceral, Thoracic, and Vascular Surgery at the University Hospital Carl Gustav Carus Dresden (Dresden, Germany) between 2005 and 2021 were used for analyses. 158 samples from Dresden were used as a discovery cohort, 175 samples from Dresden, and 135 samples from Cologne were used as validation cohort. Samples were selected sequentially from the respective biobank. To rule out sample age as a cofounding factor, correlation analysis of serum level and sample age was performed. No significant or only weak correlation was found (Figures S4G and S4H). The majority of patients were treated with gastrectomy, including transhiatal proximal gastrectomy and gastrectomy as part of a multivisceral resection (87%). The minority were treated with a subtotal gastrectomy (11%) or only in a palliative manner (2%). Patients treated in a neoadjuvant manner mostly received the FLOT protocol (72%), followed by the ECF (14%) and PLF protocol (4%), while 10% of the patients received other treatment (ECX, EOX, FLO, FLP, FOLFOX). All GC and GEJ were confirmed histologically by a trained pathologist. Serum from healthy donors (n = 82) was obtained from the Department of Visceral, Thoracic and Vascular Surgery at the University Hospital Carl Gustav Carus (Dresden, Germany) or the German Red Cross Blood Donation Service North-East. A person was considered a healthy donor (HD) if no present or past tumor disease or active disease with an immune response was known (31 % blood donors, 5 % patients with varicose veins, 23 % patients with hernias, 41 % patients with lipomas, vascular malformations, cholelithiasis, or reflux). Venous blood was collected into serum separator tubes on the day of surgery or up to 10 days before surgery or at blood donation, respectively. All samples were centrifuged (30 min to 4 hrs after collection, 12 min, 1500 x g, 4 °C), and the aliquoted serum was stored at -80 °C immediately. Only aliquots with a maximum of four freeze-thaw cycles were used. All patients and healthy donors gave written informed consent, and the study was approved by the Ethics Committee of the Technische Universität Dresden (EK76032013) and the University of Cologne (16-317), respectively. Clinical tumor stages were classified according to the tumor-node-metastasis (TNM) classification system (Union for International Cancer Control (UICC); Edition 8, stages were updated according to the pathological information if needed). Clinical characteristics are shown in Tables 1 and S3–S6. The study was performed according to the STARD and REMARK protocol.^46^^,^^47^

### Method details

#### TCGA and GTEx RNA-Seq analysis

A web service for cancer and normal gene expression profiling and interactive analyses (GEPIA, RRID:SCR_026154) was used to determine gene expression between GC and healthy controls. All data was based on the Genotype-Tissue Expression (GTEx) and the TCGA project.^48^

#### Single cell RNA-seq data processing

A dataset of 165,505 single cells from 10 patients (blood, paratumor, and tumor samples) was obtained as described in the data availability section. The FASTQ reads from the gene expression (GEX) and V(D)J sequences were aligned to the respective reference genomes (GRCh38-2024A, VDJ-GRCh38-alts-ensembl-7.1.0) using Cell Ranger 9.0.0 (RRID:SCR_017344) and the corresponding alignment pipelines for counting and V(D)J analysis. Further preprocessing and analysis were performed using Scanpy 1.10.4 (RRID:SCR_018139), which included quality control steps by filtering for cells with a low UMI count (<400 UMIs), a low gene number (<200 genes), and a high mitochondrial transcript count (>30%).^49^ Moreover, transcripts that were detected in fewer than 3 cells were excluded. Gene expression counts were normalized to the median of the total transcript count per cell in each batch to correct for differences in sequencing depth per cell. Highly expressed genes, accounting for more than 10% of the transcript count per cell, were excluded. Gene expression counts were log_2_(x+1) transformed and concatenated into an AnnData object for downstream analysis. First, doublets were removed using scDblFinder 1.16.0 (RRID:SCR_022700), and additional doublets were identified during iterative subclustering by detecting contradictory cell marker expression.^50^ Further doublet removal was performed by utilizing the V(D)J sequence to exclude T cells with more than 2 copies of TRA and TRB subunits. Cell types of interest were identified by subclustering and analyzing the expression of specific marker genes (Figures S1A, S1D, S2A, and S2F; Tables S7 and S8). Tumor cells were identified through clustering, copy number variation (CNV) analysis, and comparison with the results from K. Sun et al.^45^ Finally, after annotating the cell clusters as epithelial cells, tumor cells, CD8^+^ T cells, CD4^+^ T cells, regulatory T cells, and NK cells, the expression of *LGALS9* (Gal-9) and *HAVCR2* (TIM-3) was retrieved for each cell type.

#### Multiplex immunohistochemistry

2.5 μm thick formalin-fixed paraffin-embedded (FFPE) tissue sections were stained using the Opal™ System (Akoya Biosciences, Marlborough, Massachusetts, USA, RRID:AB_3665660) on the Ventana Discovery Ultra Platform (Ventana Medical Systems, Basal, Switzerland, RRID:SCR_021254) as previously described.^51^ In brief, following deparaffinization and rehydration, antigen retrieval was performed using Cell Conditioning (CC) Solution 1 (Ventana Medical Systems, pH 9) at 95 °C for 32 min. Subsequently, the tissues were incubated with a primary antibody, followed by incubation with an appropriate horseradish peroxidase-conjugated OmniMap secondary antibody (Ventana Medical Systems) and an Opal tyramide signal amplification (TSA) fluorophore (Akoya Biosciences). To remove antibody complexes, heat-induced stripping was performed by incubating the sections in CC2 (Ventana Medical Systems, pH 6) at 100 °C for 24 min. This staining process was repeated five times to detect six distinct markers using six different primary antibodies and Opal fluorophores. Individual antibody clones, suppliers and dilutions are listed in Table S9. After the staining cycles, tissue sections were counterstained with DAPI (Sigma-Aldrich, St. Louis, Missouri, USA) and mounted using Fluoromount-G® medium (SouthernBiotech, Birmingham, Alabama, USA). The stained slides were stored at 4 °C in the dark until imaging. Tissue imaging was performed using the PhenoImager® HT instrument (Akoya Biosciences, RRID:SCR_023772). Whole slide scans were captured at 100x magnification and regions of interest (ROIs) were identified using Phenochart™ software (Akoya Biosciences, RRID:SCR_019156). Multispectral images (MSIs) were subsequently acquired at 200x magnification, spectrally unmixed, and exported as multi-channel TIFF files using inForm software (Akoya Biosciences, RRID:SCR_019155). Representative images were processed using ImageJ software (RRID:SCR_003070).^52^

#### Serum analysis

Seventeen different soluble immune checkpoint proteins were measured using Luminex xMAP technology (RRID:SCR_018025) as described before (data for sTIM-3 shown in Figures S3A and S3B).^53^ Enzyme-linked immunosorbent assay (ELISA) was used for validation according to the manufacturer’s protocol for quantification of sTIM-3 and Gal-9 (Human TIM-3 ELISA Kit – Quantikine, Catalog #: DTIM30, Human Gal-9 ELISA Kit – Quantikine Catalog #: DGAL90, R&D Systems, Minneapolis, USA). The assay's sensitivity was 8.75 pg/ml (TIM-3) and 0.028 ng/ml (Gal-9). Varioskan LUX (Thermo Fischer, Waltham, USA, RRID:SCR_026792) was used for the readout. CEA, CA72-4, and CA19-9 values were retrieved from the patient's regular clinical blood examinations, which were conducted by the certified Institute of Laboratory Medicine at University Hospital Cologne or the University Hospital Carl Gustav Carus Dresden, respectively.

### Quantification and statistical analysis

Data is presented in dot plots with median, scatter, or box plots. Unpaired, two-tailed Student’s t-test was used for the comparison of two groups. One-way ANOVA with Tukey statistics was used for multiple comparisons. Receiver operating characteristic (ROC) in combination with Youden's J statistic was used for cut-off optimization. For survival analysis, Kaplan-Meier with Log-rank test or Cox regression was used. For optimization of the threshold between low and high serum levels for prognostic features, the median was used as a starting point and empirically improved in steps of ±0.5, 0.05, 0.005, and 0.001 ng/ml. For propensity score matching the cardinality matching method was used with a 1:1 ratio as described here.^54^ A *P*-value of < 0.05 was considered statistically significant and a confidence interval of 95 % was used when stated. Logistic and Cox regression analyses, propensity score matching, and random cohort separation were performed with R statistical software (Version 4.2.0. R Core Team, 2022. R: A language and environment for statistical computing. R Foundation for Statistical Computing, Vienna, Austria, RRID:SCR_001905). GraphPad Prism 9.0 (GraphPad Software, La Jolla, USA, RRID:SCR_002798) was used for all other plotting and analyses.
