# Supplementary material for: Soluble TIM-3 and galectin-9 predict survival in gastric and gastroesophageal junction cancer
Source: iScience. 2025 Oct 28;28(11):113871. doi: 10.1016/j.isci.2025.113871 (PMC12661199; doi:10.1016/j.isci.2025.113871)
Supplement: Document S1. Figures S1–S5 and Tables S1–S9 [file mmc1.pdf]

iScience, Volume *28*

Supplemental information

Soluble TIM-3 and galectin-9 predict survival in gastric and gastroesophageal junction cancer

David Digomann, Charlotte Reiche, Antonia Stammberger, Tido Willms, Loreen S. Rudek, Anders Grabenkamp, Anna Klimova, Jamie Kölbel, Felix Merboth, Loreen Natusch Bufe, Carolin Beer, Therés Golle, Sarah Cronjaeger, Franziska Hoffmann, Luisa Kranich, Marc Schmitz, Christiane J. Bruns, Hans A. Schlößer, Jürgen Weitz, Lena Seifert, and Adrian M. Seifert

1 SUPPLEMENTARY FIGURES

Supplementary Figure S1: Dot plots and heat maps of cell-type marker genes

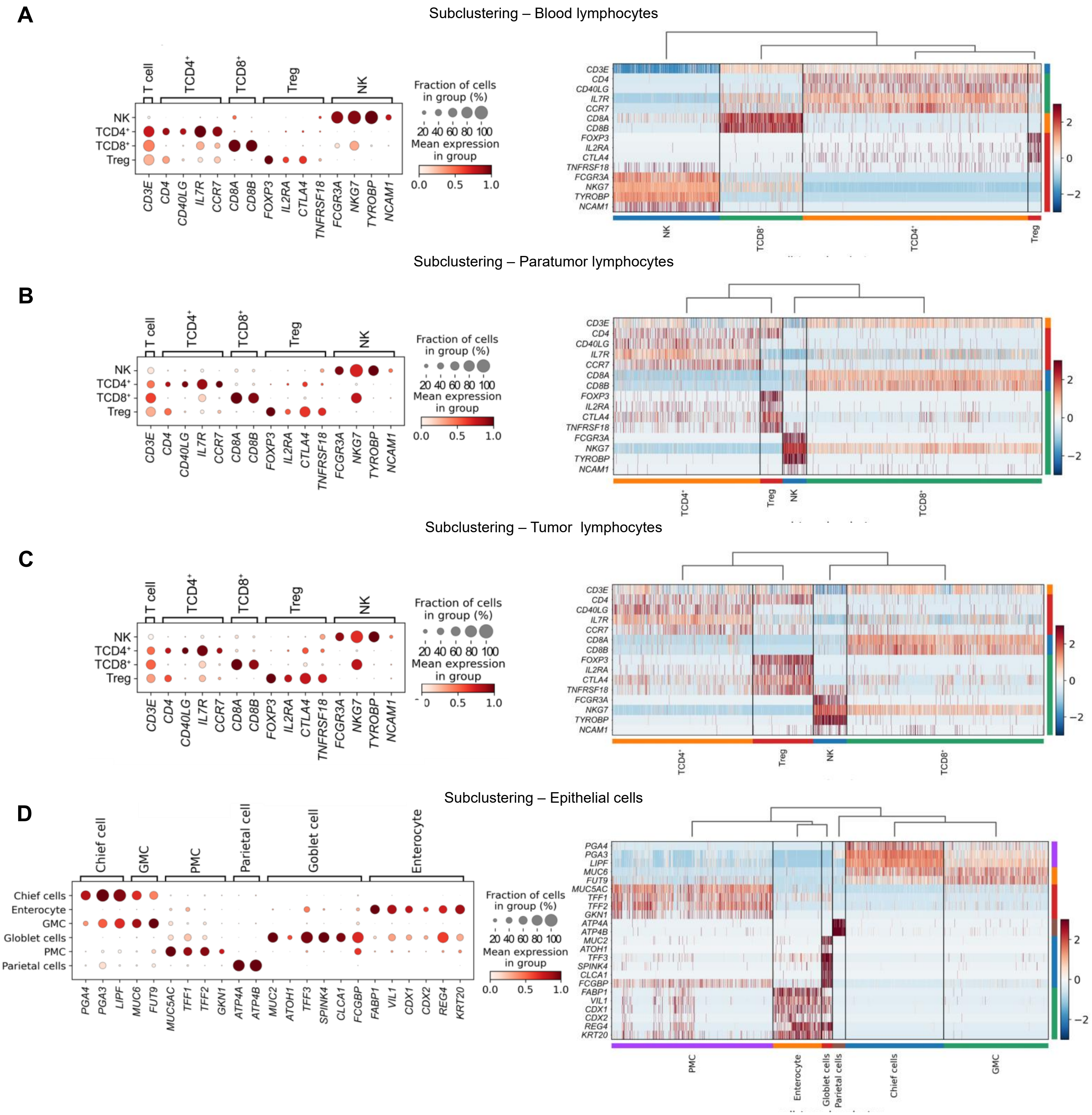

Supplementary Figure S1. Dot plots and heat maps showing cell-type marker genes for NK cell, $CD4^+$ T cell, $CD8^+$ T cell, and regulatory T cell clusters derived from scRNA-seq data **(A)** blood, **(B)** paratumor, and **(C)** tumor lymphocytes **(D)**. Dot plot and heat map with signature genes for epithelial cell clusters (chief cells, parietal cells, goblet cells, enterocytes, gastric mucous cells (GMC), and pit mucous cells (PMC)) from paratumor samples.

Supplementary Figure S2: UMAP visualizing batch distribution and dot plots with signature genes

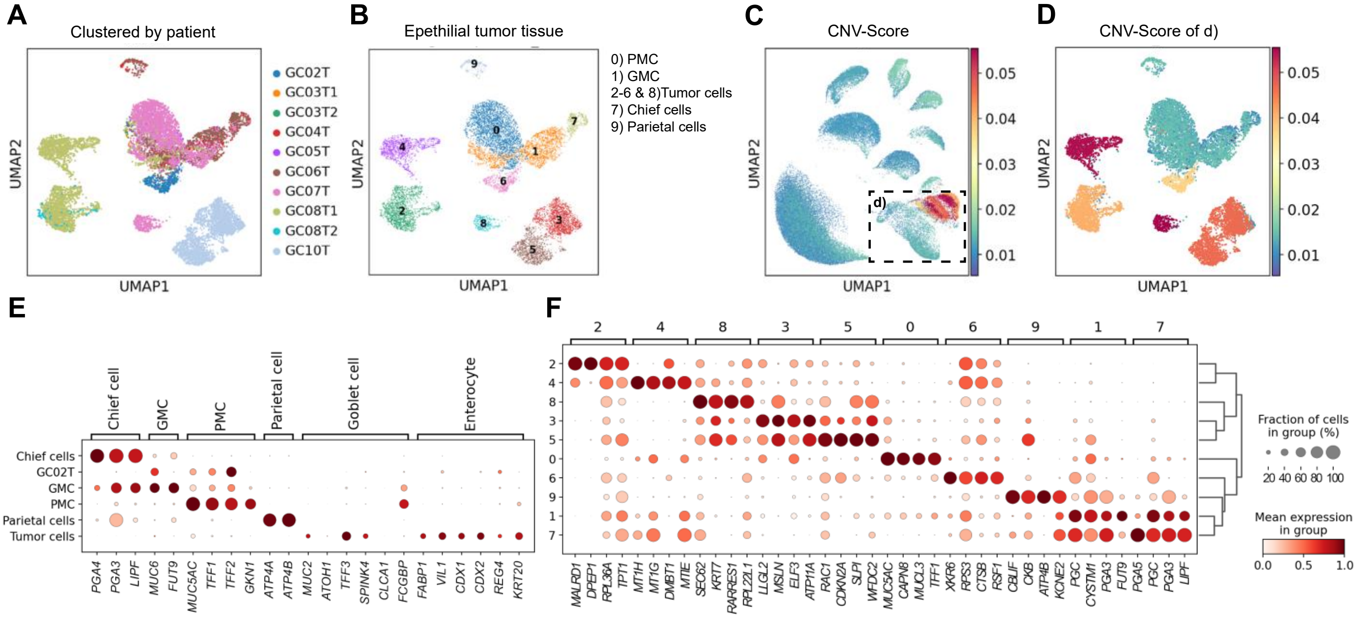

Supplementary Figure S2. UMAP visualizing batch distribution and dot plots with signature genes. **(A)** UMAP visualizing batch distribution for tumor tissue samples. **(B)** UMAP with Leiden clusters for epithelial cells and potential tumor cells from tumor tissue. **(C)** Global UMAP showing CNV counts for all cell types. **(D)** UMAP showing CNV counts for only epithelial and tumor cells. **(E)** Dot plots with signature genes for epithelial cell clusters (chief cells, parietal cells, goblet cells, enterocytes, gastric mucous cells (GMC), and pit mucous cells (PMC)) in tumor tissue samples. **(F)** Dot plots representing the top four differentially expressed genes for the Leiden clusters shown in (B).

Supplementary Figure S3: sTIM-3 level and survival association in the discovery cohort

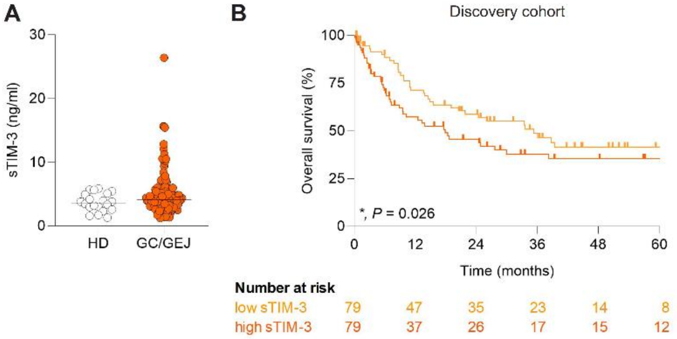

Supplementary Figure S3. sTIM-3 level and survival association in the discovery cohort. **(A)** Dot plot depicting sTIM-3 in serum from healthy donors (HD) and gastric cancer patients (GC). Mann Whitney test, *P* = 0.119. **(B)** Kaplan-Meier curve for patients with sTIM-3 above and below the median. Median survival 35.28 and 17.84 months, Log-rank *P* = 0.026.

Supplementary Figure S4: Comparison to other tumor markers and investigation of confounding factors

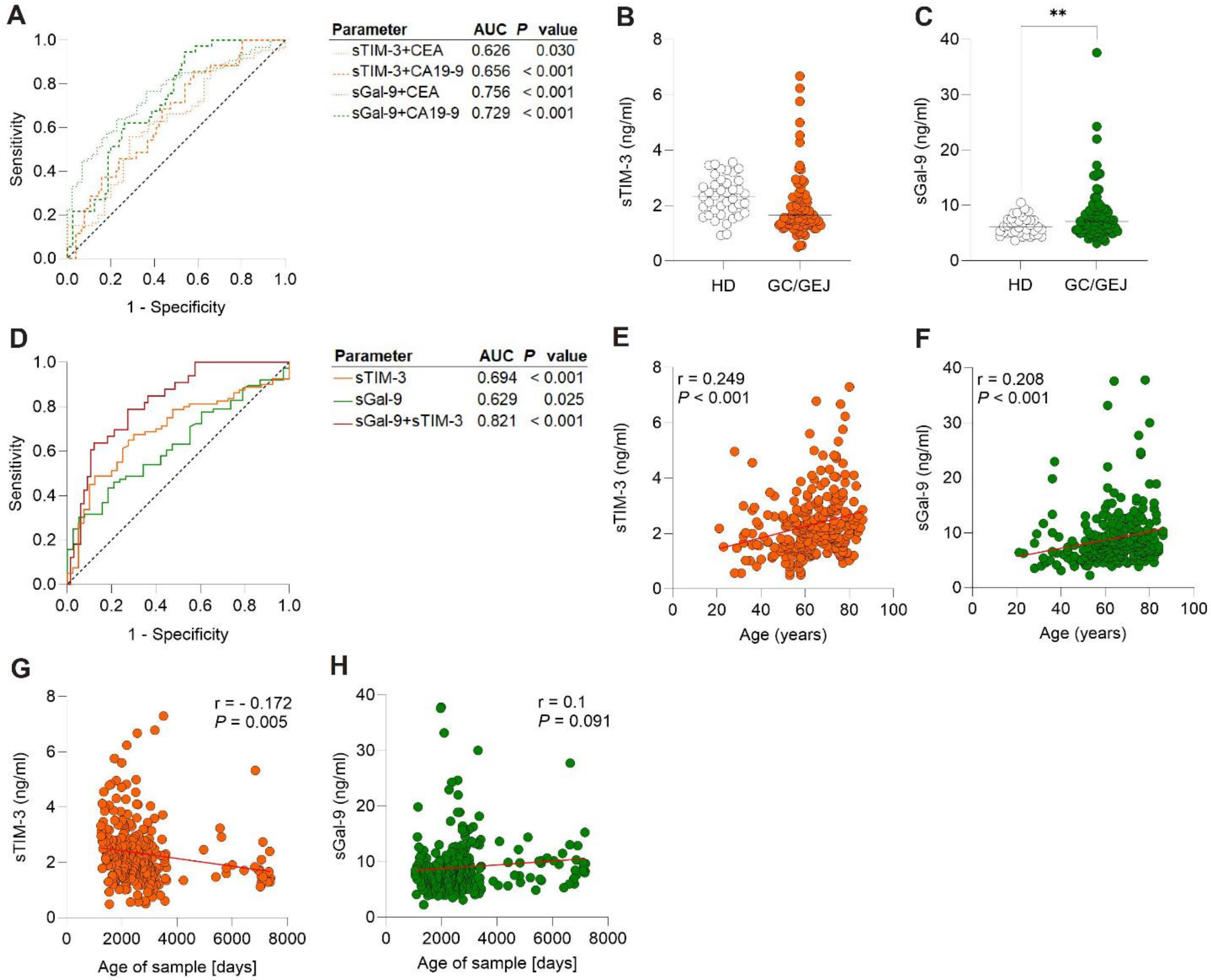

Supplementary Figure S4. Comparison to other tumor markers and investigation of confounding factors. **(A)** ROC curve of indicated combined markers based on logistic regression analysis with an AUC significantly different to chance for all combinations (sTIM-3+CEA: n = 35/86 (HD/GC); CI: 0.516-0.736; sTIM-3+CA19-9: n = 35/76 (HD/GC); CI: 0.550-0.761; sGal-9+CEA: n = 44/94 (HD/GC); CI: 0.674-0.837; sGal-9+CA19-9: n = 37/80 (HD/GC). **(B)** Serum levels of sTIM-3 from GC patients and healthy donors propensity score matched (cardinality method for matching, unpaired t-test with Welch's correction, HD vs. GC *P* = 0.05, n = 40 vs. 80). **(C)** Serum levels of sGal-9 from healthy donors and GC patients propensity score matched (cardinality method for matching, unpaired t-test with Welch's correction, HD vs. GC *P* = 0.002, n = 38 vs. 76). **(D)** ROC curve of sTIM-3, sGal-9, and sTIM-3+sGal-9 with an AUC significantly different to chance with

propensity score matched cohorts (cardinality method for matching, sTIM-3: n = 40/80 (HD/GC), CI: 0.597-0.791; sGal-9: n = 38/76 (HD/GC), CI: 0.527-0.732; sTIM-3+sGal-9: n = 33/66 (HD/GC), CI: 0.738-0.904). Scatterplot with Pearson correlation of **(E)** sTIM-3 and **(F)** sGal-9 serum levels with age (sTIM-3: n = 264; sGal-9: n = 278). Scatterplot with Pearson correlation of **(G)** sTIM-3 and **(H)** sGal-9 serum levels with sample age (sTIM-3: n = 264; sGal-9: n = 278).

Supplementary Figure S5: Survival analyses of primarily resected or neoadjuvant-treated subcohorts

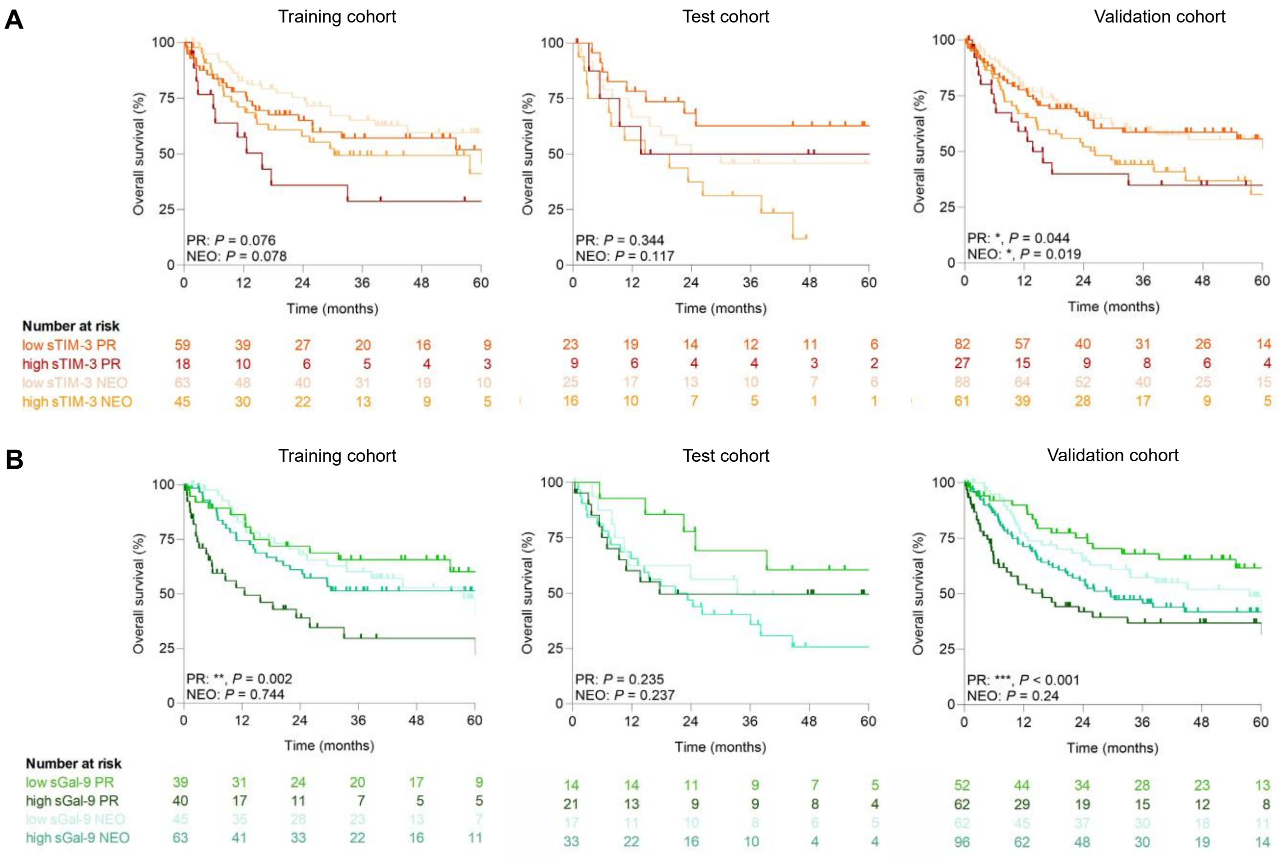

Supplementary Figure S5. Survival analyses of primarily resected or neoadjuvant-treated subcohorts. **(A)** Kaplan-Meier curves for patients with high or low sTIM-3 levels in the training, test, and validation cohort differentiated in PR and NEO subgroups. A threshold of 2.5 ng/ml for sTIM-3 was applied. Median survival of sTIM3 low vs. high in Training PR: 60.1 vs. 15.78 months, Log-rank *P* = 0.076; Training NEO: low vs. high: undefined* vs. 30.35 months, Log-rank *P* = 0.078. Test PR: undefined* vs. 36.92 months, Log-rank *P* = 0.344; Test NEO: 26.93 vs. 17.02 months, Log-rank *P* = 0.117. Validation PR: undefined* vs. 13.73 months, Log-rank *P* = 0.044; Validation NEO: undefined* vs. 26.27 months, Log-rank *P* = 0.019. **(B)** Kaplan-Meier curves of patients with high or low sGal-9 levels in the training, test, and validation cohort differentiated in PR and NEO subgroups. A threshold of 7.2 ng/ml for sGal-9 was applied. (Median survival of sGal-9 low vs. high in Training PR: undefined* vs. 12.52 months, Log-rank *P* = 0.002; Training NEO: low vs. high: 57.7 vs. undefined* months, Log-rank *P* = 0.744. Test PR: undefined* vs. 17.64 months, Log-rank *P* = 0.235; Test NEO: 33.38 vs. 22.09 months, Log-rank *P* = 0.237. Validation PR: undefined* vs. 15.78

months, Log-rank $P < 0.001$; Validation NEO: 57.7 vs. 29.52 months, Log-rank $P = 0.24$. _*probability of survival exceeds 50 % at the longest time point_.

SUPPLEMENTARY TABLES

Supplementary Table S1: sGal-9 as tumor marker (test cohort)

	**Predicted +**	**Predicted -**
**GC**	72.22 %<br>True positive rate	27.78 %<br>False negative rate
**HD**	35.14 %<br>False positive rate	64.86 %<br>True negative rate

Supplementary Table S2: sGal-9+sTIM-3 as tumor marker (validation cohort)

	Predicted +	Predicted -
**GC**	91.84 %<br>True positive rate	8.16 %<br>False negative rate
**HD**	58.54 %<br>False positive rate	41.46 %<br>True negative rate

Supplementary Table S3: Clinicopathologic characteristics of the discovery cohort

	n	(%)
**Mean age**	65.84 (years)	
**Sex**		
Female	54	(34.18)
Male	104	(65.82)
**T Stage**		
0	6	(3.8)
1	33	(20.89)
2	23	(14.56)
3	56	(35.44)
4	29	(18.35)
Unknown	11	(6.96)
**N Stage**		
0	61	(38.61)
1	24	(15.19)
2	24	(15.19)
3	38	(24.05)
Unknown	11	(6.96)
**M Stage**		
0	125	(79.11)
1	33	(20.89)
**UICC Stage**		
CR	5	(3.16)
I	44	(27.85)
II	30	(18.99)
III	45	(28.48)
IV	34	(21.52)
**Neoadjuvant Treatment**		
Yes	84	(53.16)
No	73	(46.2)
Unknown	1	(0.64)

Supplementary Table S4. Clinicopathologic characteristics of HD

**Serum level**	**PR, n (%)**		**HD, n (%)**		***P*-value***
**Mean age**	68.63 (years)		52.78 (years)		*****, <0.001[a]**
**Sex**					
Female	49	(38.28)	29	(35.37)	0.77[b]
Male	79	(61.72)	53	(64.63)	

*compared to PR

[a] t-test

[b] Fisher's exact test

Supplementary Table S5: Clinicopathologic characteristics of the validation cohort

**Cohort**	**sTIM-3 low, n (%)**		**sTIM-3 high, n (%)**		***P*-value**
**Total**	174		90		
**Mean age**	61.35 (years)		66.36 (years)		****, 0.005[a]**
**Sex**					
Female	74	(42.53)	34	(37.78)	0.51[b]
Male	100	(57.47)	56	(62.22)	
**Tumor localization**					
GEJ	16	(9.2)	10	(11.11)	0.665[b]
Stomach	158	(90.8)	80	(88.89)	
**T Stage**					
0	7	(4.02)	8	(8.89)	***, 0.013[c]**
1	39	(22.41)	13	(14.45)	
2	23	(13.22)	4	(4.44)	
3	60	(34.49)	31	(34.45)	
4	36	(20.69)	30	(33.33)	
Unknown	9	(5.17)	4	(4.44)	
**N Stage**					
0	71	(40.8)	34	(37.78)	0.429[c]
1	30	(17.24)	11	(12.22)	
2	21	(12.07)	16	(17.78)	
3	42	(24.14)	25	(27.78)	
Unknown	10	(5.75)	4	(4.44)	
**M Stage**					
0	151	(86.78)	68	(75.56)	***, 0.025[b]**
1	23	(13.22)	22	(24.44)	
**UICC Stage**					
CR	9	(5.17)	8	(8.89)	***, 0.049[c]**
I	49	(28.16)	15	(16.67)	
II	43	(24.71)	18	(20)	
III	50	(28.74)	27	(30)	
IV	23	(13.22)	22	(24.44)	
**Neoadjuvant Treatment**					
Yes	89	(51.15)	62	(68.89)	****, 0.008[b]**
No	83	(47.7)	28	(31.11)	
Unknown	2	(1.15)	0	(0)	

[a] t-test

[b] Fisher's exact test

[c] Chi-squared test

Supplementary Table S6: Clinicopathologic characteristics of the validation cohort

Cohort	sGal-9 low, n (%)		sGal-9 high, n (%)		*P*-value
**Total**	117		161		
**Mean age**	60.03 (years)		66.2 (years)		*****, <0.001[a]**
**Sex**					
Female	53	(45.3)	59	(36.65)	0.173[b]
Male	64	(54.7)	102	(63.35)	
**Tumor localization**					
GEJ	7	(5.98)	20	(12.42)	0.1[b]
Stomach	110	(94.02)	141	(87.58)	
**T Stage**					
0	3	(2.56)	13	(8.07)	0.084[c]
1	30	(25.64)	28	(17.39)	
2	15	(12.82)	15	(9.32)	
3	35	(29.92)	61	(37.89)	
4	31	(26.5)	36	(22.36)	
Unknown	3	(2.56)	8	(4.97)	
**N Stage**					
0	50	(42.74)	64	(39.75)	0.901[c]
1	17	(14.53)	25	(15.53)	
2	19	(16.24)	22	(13.66)	
3	28	(23.93)	42	(26.09)	
Unknown	3	(2.56)	8	(4.97)	
**M Stage**					
0	104	(88.89)	130	(80.75)	0.07[b]
1	13	(11.11)	31	(19.25)	
**UICC Stage**					
CR	5	(4.27)	12	(7.45)	0.147[c]
I	35	(29.91)	36	(22.36)	
II	29	(24.79)	30	(18.63)	
III	35	(29.92)	52	(32.3)	
IV	13	(11.11)	31	(19.26)	
**Neoadjuvant Treatment**					
Yes	62	(52.99)	97	(60.25)	0.24[b]
No	53	(45.3)	62	(38.51)	
Unknown	2	(1.71)	2	(1.24)	

[a] t-test

[b] Fisher's exact test

[c] Chi-squared test

58

Supplementary Table S7: Cell signature genes for main cell types

Cell types	Signature genes
B cell	MS4A1, CD19, BANK1, VPREB3, CD79A
Plasma cell	JCHAIN, IGKC, MZB1, DERL3, CD79A
Endocrine cell	CHGA, PCSK1N, TTR, SCG3, SCG5, EPCAM, KRT18, KRT19
Epithelial cell	MUC5AC, MUC1, S100P, LIPF, TFF1, TFF2, PSCA, EPCAM, KRT18, KRT19
T & NK cells	CD2, CD3E, CD3D, CD3G, CD7
Erythrocytes	HBB, HBA1, ALAS2, HBA2, CA1
Mast cells	TPSAB1, TPSB2, CPA3, TPSD1, GATA2
Myeloid cell	AIF1, CD68, CD14, FCN1, S100A9, MS4A7
Endothelial cell	PLVAP, VWF, PECAM1, ACKR1, CLDN5, CD34
Fibroblasts	PDGFRA, PDPN, DCN, DPT, TRPA1
Smooth muscle cells	ACTA2, ACTG2, MYH11, RGS5, NDUFA4L2

Supplementary Table S8. Cell signature genes for epithelial cells

Cell types	Signature genes
Chief cell	PGA4, PGA3, LIPF
Basal Gland mucous cell (GMC) or mucous neck cell	MUC6, FUT9
Pit mucouse cell (PMC) (Surface mucous cell)	MUC5AC, TFF1, TFF2, GKN1
Parietal cell	ATP4A, ATP4B, GIF
Goblet cell	MUC2, ATOH1, TFF3, SPINK4, CLCA1, FCGBP
Enterocyte	FABP1, VIL1, CDX1, CDX2, REG4, KRT20

Supplementary Table S9. List of antibodies and applied settings for the multiplex immunohistochemistry staining

Antibody	Dilution	Supplier	Incubation	Clone	RRID	Opal	Dilution
Anti-CD4	1:50	Abcam plc.	36°C, 32min	EPR6855	AB_2750883	650	1:150
Anti-CD8	prediluted	Ventana Medical Systems, Inc.	36°C, 32min	SP57	AB_2335985	540	1:300
Anti-FoxP3	1:50	Abcam plc.	36°C, 32min	A236A/E7	AB_445284	520	1:50
Anti-Gal-9	1:50	Cell Signaling Technology, Inc.	36°C, 32min	D9R4A	AB_2799456	570	1:50
Anti-PanCK	prediluted	Ventana Medical Systems, Inc.	36°C, 32min	AE1/AE3/PCK26	AB_2810237	690	1:50
Anti-TIM-3	1:50	Cell Signaling Technology, Inc.	36°C, 60min	D5D5R	AB_2716862	620	1:50
